# The Surgical Diagnosis of Right-Side Abdominal Diseases1Read at the meeting of the Society of Physicians, Canandaigua, N. Y., February 7, 1901.

**Published:** 1901-03

**Authors:** A. L. Beahan

**Affiliations:** Canandaigua, N. Y.


### THE SURGICAL DIAGNOSIS OF RIGHT-SIDE ABDOMINAL DISEASES.[1]

By A. L. BEAHAN, M. D., Canandaigua, N. Y.

A GROUP of recent cases of the right abdominal region calls attention to the interesting features of diagnosis of diseases common to that locality. This section presents more varied and common diseases than any other of the body. In its topography are included the right lobe of liver, the gall-bladder, duodenum, pancreas, hepatic flexures of the colon, right kidney and its suprarenal capsule, cecum and ascending colon, coils of small intestines, appendix and right ureter, and it is bounded posteriorly in part by the right psoas muscle, an important fact.

The most important organs, because of frequency of involvement in disease, are the appendix and gall-bladder in the male, tube and ovary in the female, while the kidney of this corresponding right side, is very often surgically diseased. A specific and differential diagnosis is demanded, beginning with one of the more frequent diseased organs, the gall-bladder.

Acute inflammation of the gall bladder manifests itself somewhat suddenly, with pain on right side of the abdomen, rapidly becoming general. A rapid and feeble pulse, quick thoracic breathing, fever, intense depression, marked tenderness on pressure, especially over the right side of the abdomen, rapidly developing tympanitis, persistent vomiting and an extremely anxious expression of countenance, are usually present. The acute peritonitis, localised at first, becomes general.

In all gall-bladder inflammations there is a tender spot, it may be called the "Mayo Robinson point," at the juncture of the outer one-third with the inner two-thirds of a line passing from the umbilicus to the ninth rib, at the notch found in the line of the costal margin.

---

1. Read at the meeting of the Society of Physicians, Canandaigua, N. Y., February 7, 1901.

For the appendix, let the same imaginary line pass from the umbilicus to the anterior superior spine of the ilium, and at the juncture of the outer third with the inner two-thirds of this line, will be found the "McBurney point." In the female it is somewhat lower.

Neither of these points are the points of tenderness in cholecystitis and appendicitis exclusively, but they serve to fix the mind on two important points in a clear manner.

The right kidney projects one-half of its size below the costal border of the right lower rib. When the kidney is enlarged from disease or a perinephritic abscess, it projects forward as a more or less smooth mass, between a possible abscess of gall-bladder or appendix. It is never movable if a perinephritic abscess binds with adhesions posteriorly.

The right ovary and tube can be recognised by conjoined vaginal and abdominal palpation, when proper care is exercised. The diagnosis of disease of tube and ovary should be possible to every practitioner of medicine and surgery, as well as to the gynecologist, to the end that uterine tinkering in ovarian disease may not be practised, as any latent ovarian disease is easily excited or increased and the uterine mucosa is of itself but little influenced by ordinary local treatment, such as cleaning and applications.

Gall-bladder troubles of common character are from calculi in its cavity, its cystic duct, or in the common, or hepatic ducts. If calculi are in either the hepatic or common duct, jaundice occurs; if in the cystic duct jaundice is not present. An enlarged gall-bladder may result from calculi, hydrops of the organ, empyema of the gall-bladder, or from a tumor essential to itself. A floating kidney often simulates gall-bladder disease, but the kidney can be pressed back into its original place, while the gall-bladder is less freely moved.

I have seen one gall-bladder filled with calculi that could be depressed below the umbilicus, making differential diagnosis uncertain, as jaundice had not occurred, because the calculi were of small size and could pass readily through the cystic and common ducts. But the appendix most taxes diagnostic skill. No symptoms are certain or peculiar to its manifestations of disease. No means of diagnosis are comfortably safe. The masters of the appendix art cannot impart exact rules. Leading symptoms are important, if present; equally important, if absent.

A recent case in a nurse we had watched a year, having a year previously treated her for rectal fissure. Only two symptoms at first were pointers,—one, menstrual pains growing worse, another constipation. You may say these are common to women. The patient at the menstrual epoch had a chill and belly pains, not so severe as to prevent caring for her patient night and day at the Natural Science Camp, Canandaigua Lake. After this no symptoms followed, except slight backache, malaise and vomiting, if cathartics were employed to relieve her constipation. The succeeding menstruation gave epigastric, followed by right inguinal, pain; then a cold spell, but not a distinct chill, followed by a temperature not above 101 1-5°, but never quite normal.

We often found 99 1-5° to be the appendical mean temperature. The pulse was never above 80. The menstrual pain, as presumed by patient, was a trifle more severe than that at the last time, and was ascribed to the fact that at the camp during the previous menstruation she might have taken cold. To increasing menstrual pains and constipation we could add but little history, not more than many women suffer, and if not in the hospital, I question if this attack would have been properly catalogued.

The bowels were freely moved, and palpation discovered a swollen and tender appendix. Operation was consented to and an appendix found swollen, with a phlegmon of meso-appendix of considerable size, so dark grey in color and so nearly boggy in feel, that the fear of its breaking down led to suturing it to the line of incision, so that if abscess developed it would be within easy reach; and for forty-eight hours our mind did not rest easy because of danger of sepsis. Two grape seeds were found in the appendix; one had much surrounding ulceration, feeding the phlegmon in meso-appendix with sepsis.

Two recent cases of appendicitis illustrate another puzzle in diagnosis. Both had attacks of peritonitis, one six weeks and the other twelve weeks previously. In either case the appendix was not to be palpated until the bowels were freely purged, and this was because in each case the appendix was posterior to the cecum, being sharply flexed on the cecum. The distension of the cecum prevented palpation of the appendix. The appendix may also be so badly disorganised as to be undiscoverable. The relation of menstrual irregularities and appendicitis seems to us to be intimate and to bear further research.

Usually the swollen appendix can be felt. With the patient in the dorsal position, legs well flexed, fingers of left hand applied to back of fingers of right hand, that the pressure may come from the left, and that with the right, so accustomed to sensitive touch, pressure may be made carefully and steadily against the psoas muscle during respiration. If the rectus comes up the hard pressure must be under and at the side of the muscle. A hard right rectus means appendicitis. If it continues after the patient is otherwise relaxed under an anesthetic, it means that pus is present. Painful micturition means that the appendix lies pointing toward the bladder. Pain in the back means a backward and upward pointing of appendix.

Most groin abscesses are from the appendix. Dr. Joseph Price recently asked the internes of several Philadelphia hospitals how many psoas abscesses they had seen in their terms of service. All answered none.

Perinephritic abscess pushes the kidney forward and in a recent case the diagnosis was made correctly, as proven by operation. The kidney was well forward and, though much enlarged by the inflammatory process, continued to give its smooth normal contour. The incision for abscess was made posteriorly. If made over prominence of swelling, to reach the abscess would require incision through the kidney, or a nephrectomy, a mistake recently observed, the kidney being needlessly sacrificed. In no other locality is a refinement in diagnostic acumen and ability more necessary. In no other does a similar gratification occur. The touch aids the judgment, the mind weighs evidence, and a correct diagnosis results.

18 Gorham Street.

